# Dragon Fruit Peel Extract Enriched-Biocomposite Wrapping Film: Characterization and Application on Coconut Milk Candy

**DOI:** 10.3390/polym15020404

**Published:** 2023-01-12

**Authors:** Wantida Homthawornchoo, Nur Fairuza Syahira Mohamad Hakimi, Orapan Romruen, Saroat Rawdkuen

**Affiliations:** 1 Innovative Food Packaging and Biomaterials Unit, School of Agro-Industry, Mae Fah Luang University, Chiang Rai 57100, Thailand; 2 Food Science and Technology Program, School of Agro-Industry, Mae Fah Luang University, Chiang Rai 57100, Thailand; 3 Food Sciences and Technology Program, School of Applied Science, Universiti Teknologi MARA, Shah Alam 45100, Malaysia

**Keywords:** active film, dragon fruit peel extract, rice starch-based film, antioxidant, lipid oxidation

## Abstract

Bio-based film is an eco-friendly alternative to petroleum-based packaging film. The effects of biocomposite wrapping film enhanced with dragon fruit peel extract (0, 2% *w*/*v*, respectively) and currently used commercial packaging film (polypropylene; PP) on coconut milk caramels during storage (30 °C, 75% RH, nine days) were studied. Both 0% and 2% DPE-enriched biocomposite films were thicker and had higher water vapor permeability and solubility than the PP film but poorer mechanical characteristics. In addition, the 2% film possessed antioxidants and antioxidant ability. A FESEM micrograph revealed the rough surface and porous path of the biocomposite films. Over the storage time, the moisture content, water activity, and springiness of the coconut milk caramel candy wrapped in the PP and all DPE-enriched biocomposite films were not significantly altered. However, the lipid oxidation as the thiobarbituric acid reactive substance (TBARS) and hardness of all coconut caramels were significantly (*p* < 0.05) increased during storage. Furthermore, the hardness of coconut candy covered in the control (0% DPE) biocomposite film was more pronounced on day nine of storage. However, the changes in quality characteristics of the coconut candy wrapped in each film type need to be better established. The investigating factors influencing the quality deterioration of coconut milk candy should be further identified to mitigate their effects and extend the shelf-life of the coconut candy.

## 1. Introduction

Bio-based packaging has been gaining more attention in the food packaging industry these days. Using bio-based packaging is portrayed as an alternative environmentally friendly packaging [1]. In addition, consumers are now eager to support food products that are aware of the sustainable living lifestyle [2]. Therefore, biocomposite packaging films have been widely applied on several food product types, using various biopolymers [3], rice [1], corn [4], soy protein isolate [5], gelatin [6], chitosan [7], polylactic acid [8], etc. However, the application of bio-based food packaging must ensure the safety and quality of food products [9,10]. Ideal bio-based food packaging should be as good as synthetic packagings such as polyethylene [11,12] and polypropylene [13], having a superior ability in preserving the quality and extending the shelf-life of a food product.

The primary difficulties of the bio-based packaging film, such as low cost-efficiency and low degradation rate, have limited their implementation as food packaging [1]. At the same time, rice starch-based packaging film has been a promising alternative to biopolymer packaging due to its abundantly available nature, low cost, and good film-forming ability [14,15]. However, rice starch-based biopolymer films possess poor mechanical properties and high hygroscopicity [16,17].

Various approaches to enhance the functional and physicochemical properties [18] of rice starch-based films have been investigated, e.g., the addition of copolymer blends [19] such as gelatin [20], chitosan [21], pectin [22], reinforcement with the cellulose fiber [23], and incorporation of active ingredients [14] to form an active packaging film. In this study, the addition of pectin in a rice starch-based film as a copolymer to improve its mechanical properties was investigated. Pectin is a polysaccharide obtained from agricultural by-products [24]. Pectin enhances the ability of the biopolymer matrix to carry the bioactive compound [25], promotes a film-forming ability, and improves the mechanical properties of the resulting film.

As a safer alternative to its synthetic counterpart, incorporating a natural active ingredient or extract derived from agricultural materials and by-products into the biocomposite films, such as curcumin [14,20], nanocellulose [16], catechin-Kradon extract [26], mango peel extract [27], dragon fruit peel extract [28] or, when used as metal surface protection, Chinese yam peel extract [29] or purple passion fruit peel extract [30], seems to have become widely attractive. Among the agricultural by-products, dragon fruit peel is one of the natural sources of active ingredients. Dragon fruit peel extract (DPE) possesses phenolic compounds and betacyanins [31,32,33,34]. The application of DPE (20–40% *w*/*w*) as a natural antioxidant in beef sausage was found to delay lipid oxidation in the resulting product [35]. However, in food packaging, DPE investigation remained restricted.

Coconut milk candy is a caramel confectionery that is sweet, sticky, and chewy. Typically, the candy is prepared by gradually heating coconut milk, glutinous rice flour, and palm sugar [36,37]. It is known as Kalamae in Thailand, *Dodol* in Malaysia, Indonesia, Singapore, and Brunei, and *Kalama* in Myanmar [38,39]. A gooey, luscious, and firm texture is preferable for consumer acceptance [40]. Numerous factors influencing the quality and shelf-life of coconut milk candy, including the cooking temperature, heating time, humectants, sugar quantity, and types of sugar, have been investigated to improve the texture of the developed coconut caramel candy [37,39,40,41,42,43] to be soft and chewy but have low stickiness. Nevertheless, despite the processing conditions, a deterioration in quality, particularly texture hardening, was still evident during storage, resulting in coconut milk candy having a short shelf life of 7 to 14 days [39,40]. There have been attempts to address the storage-induced hardening problem in coconut candy, e.g., applying amylase enzyme [44,45] and heat treatment [39] to improve and retain a desirable texture and extend the shelf-life of the resulting caramel candy. However, some limitations of the proposed method have restricted its utilization in a wider context. Thus, alternative active packaging solutions to the quality deterioration problem in coconut milk candy are still required.

Typically, polypropylene (PP) film was used to wrap coconut milk candies. However, the synthetic nature of the material reduces its appeal as a candy wrapper in light of consumers’ concern for the environment. At the same time, information regarding the use of bio-based active film as packaging for coconut milk candy is still limited. Concerning the deterioration of the coconut milk candy’s quality and environmental sustainability, it is necessary to investigate the application of the bio-based active film to the coconut milk candy to preserve the quality of the coconut milk candy in the most environmentally friendly manner. Therefore, this study aimed to (i) characterize the biocomposite films with and without DPE integration, in comparison to a commercial PP film, and (ii) analyze the influence of the DPE-enriched biocomposite film on changes in the quality of coconut milk candy in contrast to PP film.

## 2. Materials and Methods

### 2.1. Materials

Native rice starch was acquired from Thai Flour Industry Co., Ltd., Bangkok, Thailand. Dragon fruits (*Hylocereus undatus*) were purchased from the local markets in Chiang Rai, Thailand. Coconut milk candy was obtained from Kalamae Chiang Kham community enterprise (Phayao, Thailand). The Folin–Ciocalteu’s phenol reagent and gallic acid (≥99%) were purchased from Fluka (Buchs, Switzerland). DPPH (2,2-diphenyl-1-picrylhydrazyl), and TPTZ (2,4,6-tripyridyl-s-triazine), were purchased from Sigma-Aldrich (St. Louis, MO, USA). All other chemicals and reagents used in this research were of analytical grades.

### 2.2. Preparation of Dragon Fruit Peel Extract

The white dragon fruits (*H. undatus*) were cleansed with tap water. The peels were separated, cut into small pieces (i.e., 2 cm × 2 cm), and dried at 45 °C for 24 h. The dried peels were pulverized into powder, passed through a 35-mesh sieve (500 μm), and kept in the sealed bags at a cool, dry place until further use.

The dried dragon fruit powder was extracted according to the method of Fathordoobady et al. [46]. In brief, the dried powder was extracted with the co-solvent of ethanol (95% *v*/*v*) to distilled water at a ratio of 50:50 (*v*/*v*) and stirred at 300 rpm for 20 min at room temperature. A ratio of 10 mL/g of co-solvent to dried powder ratio was applied. The mixture was subsequently filtered through Whatman paper No. 4 to remove the spent dragon fruit powder. The filtrate was centrifuged at 10,000 rpm for 15 min. The collected supernatant was concentrated using a rotary evaporator (115 VAC, Cole-Parmer, Vernon Hills, IL, USA) at 40 °C and subjected to freeze drying (Delta 2-24 LSCplus, Martin Christ Gefriertrocknungsanlagen GmbH, Osterode am Harz, Germany). The obtained dragon fruit peel extract (DPE) powder was stored at −18 °C until needed.

### 2.3. Preparation of the DPE-Enriched Biocomposite Film

The air-dried casting method was used to prepare the DPE-enriched biocomposite film according to Kaewprachu et al. [26] with slight modification. Briefly, a blend of rice starch and high methoxyl pectin at a ratio of 1:1 was used at 4.0% solid *w*/*v*, as the film-forming solution (FFS) base together with glycerol of 30% *w*/*w* solid. After heating the FFS at 85 °C for 30 min, 2.0% *w*/*v* of the dragon fruit peel extract (DPE) was incorporated into the FFS. The FFS without DPE was used as the control film. Four grams of the prepared FFS was cast on the silicone-rimmed mold, 50 mm × 50 mm in dimensions, and dried for 24 h at room temperature. Prior to further use, the dried films were conditioned at 50 ± 5% relative humidity (RH), 25 ± 0.5 °C for 48 h using a humidity-controlled cabinet (AH-80, Patron, Taichung City, Taiwan).

### 2.4. Film Property Determination

#### 2.4.1. Film Thickness

The thickness of the 0.0% (control, 0%) and 2.0% DPE (2%) biocomposite films and the commercial polypropylene (PP) film were measured at nine random locations of the five film specimens for each film type. A micrometer (Mitutoyo Corporation, Tokyo, Japan) was used to determine the thickness of the films.

#### 2.4.2. Film Appearance and Color

The appearance and color of all film samples were captured by a spectrophotometer CM-600d (Konica Minolta, Inc., Tokyo, Japan). The color values were expressed in the CIE system. Prior to measurement, a standard white tile (L* = 97.55, a* = −0.03, and b* = 1.73) was used to calibrate the colorimeter. The average values of the brightness (L*), redness (a*), yellowness (b*), and color difference (ΔE*) were obtained from measurements of three film samples.

#### 2.4.3. Film Solubility

The film solubility (FS) of the film sample was measured in triplicate according to the method of Kaewprachu et al. [26]. The FS was given as a percentage of dry weight loss relative to the film’s initial dry weight.

#### 2.4.4. Mechanical Properties

A Universal Testing Machine (Lloyd Instruments Ltd., Fareham, Hampshire, UK) was used to assess the tensile strength (TS) and elongation at break (EAB) according to ASTM D-882 [47]. Film specimens of dimensions 2 cm × 5 cm were prepared. The initial grip length was 30 mm, and the crosshead speed was 30 mm/min. Ten film samples were evaluated for each film treatment.

#### 2.4.5. Water Vapor Permeability

The water vapor permeability (WVP) was measured according to a modified ASTM E96-95 standard [48]. Briefly, the film sample was used as a cover for a silica gel-filled (0% RH) WVP cup. The WVP cup was then sealed and placed in a dry cabinet (AH-80, Patron, Taichung, Taiwan) at 25 °C, 50% RH. Each hour for eight hours, the weight of the cup was recorded. The film’s WVP was expressed in units of g∙m/m^2^ s∙Pa. For each film treatment, the WVP test was conducted in triplicate.

#### 2.4.6. Determination of Total Phenolic Content and Total Betacyanins Content

The extract solution of all film samples was prepared in advance for analysis of total phenolic content (TPC), DPPH radical scavenging activity (DPPH), ferric-reducing antioxidant power (FRAP), and total betacyanins content (TBC). Briefly, a 25 mg film sample was submerged in 5 mL of deionized water and shaken at 250 rpm at 25 °C for three h. After centrifuging at 3000× *g* for 10 min, the supernatant was collected for further analysis.

TPC was measured using the Folin–Ciocalteu assay [26] in triplicate. The standard curve of gallic acid 20–100 µg/mL was created. The TPC was expressed as mg gallic acid equivalent (GAE) per g dried film.

TBC of the film sample containing 2.0% DPE was determined in triplicate using the spectrophotometric method (Genesys 10S UV-Vis, Madison, WI, USA) according to Tenore et al. [49] with slight modifications. Briefly, the film extract solution obtained as previously described was diluted with McIlvaine buffer (pH 6.5) to obtain an absorbance of 0.8–1.0 at 538 nm. McIlvaine buffer is a mixture of 0.1 M citric acid (29.65 mL) and 0.2 M sodium phosphate dibasic (70.35 mL). The absorbance of the sample mixture at 600 nm was used to get the correct absorbance values (A). The TBC value was calculated according to Equation (1) and expressed as betanin equivalents (mg/g dried film).

(1) $$\mathrm{TBC}\;(\mathrm{mg}/L)=\frac{\left(A_{538}\times \mathrm{DF}\times \mathrm{Mw}\times 1000\right)}{\varepsilon \times L}$$

where A_538_ = corrected absorption at 538 nm

DF = dilution factor 

Mw = molecular weight of betanin (550 g/mol)

ε = molar extinction coefficient of betanin (60,000 L.∙mol^−1^∙cm^−1^)

L = path length of the cuvette (1.0 cm)

#### 2.4.7. Determination of DPPH Radical Scavenging Activity

The ability of the film samples to scavenge the DPPH radical was studied in triplicate [26,50]. A Trolox of 10–60 μM was used to develop a standard curve. Briefly, 0.15 mM DPPH in 95% ethanolic solution was added into 1.5 mL of the film extract solution. The mixture was kept in the dark for 30 min before measuring the absorbance at 517 nm with a UV-Vis spectrophotometer (Genesys 10S UV-Vis, Madison, WI, USA). A 95% methanol sample was used as a blank. The data were expressed as µmol Trolox equivalents per gram of dried film.

#### 2.4.8. Determination of Ferric Reducing Antioxidant Power (FRAP)

The method of Benzie and strain (1996) [51] with some modifications was used to determine the ferric reducing antioxidant power (FRAP) of the film samples in triplicate. Briefly, 150 μL of the film extract solution was mixed with the FRAP working reagent (2850 µL), which was a mixture at a ratio of 10:1:20 of 300 mM acetate buffer (pH 3.6), 10 m TPTZ (2,4,6-tripyridyl-s-triazine) in 40 mM HCl solution, and 20 mM FeCl_3_∙6H_2_O solution. After incubating in a 37 °C water bath in the dark for 30 min, the absorbance was measured at 593 nm (Spectrophotometer, Genesys 10S UV-Vis, Madison, WI, USA). The standard curve of 0–1000 μM Ferrous sulfate (Fe(II)) was constructed. The FRAP values were expressed as µmol Ferrous sulfate (Fe(II)) equivalents/g dried film.

#### 2.4.9. Film Morphology

A field emission scanning electron microscope (FESEM) (TESCAN MIRA, TESCAN, Brno, Czech Republic) was used to investigate the film morphology. The examinations were conducted at magnifications of 500× and 2000× for surface analysis, and 2000× for cross-sectional analysis using a 10 kV acceleration voltage.

### 2.5. Film Application to Coconut Milk Candy

The sweet coconut milk candy (10 g each) was wrapped in the current commercial packaging film (i.e., polypropylene (PP)), and the developed biocomposite films of 0% DPE (without DPE), and 2% DPE, respectively. The wrapped candies were stored at 30 ± 1 °C, 75% RH for nine days in the climatic-controlled chamber (HPP750, Memmert GmbH, Schwabach, Germany). The quality characteristics of the candy samples were determined in triplicate at day 0, 1, 3, 5, 7, and 9, respectively.

### 2.6. Determination of Quality Attributes of Coconut Milk Candy

#### 2.6.1. Moisture Content and Water Activity

The moisture content (MC) of the coconut milk candy was analyzed in triplicate according to AOAC 920.151 [52]. In brief, the sample was dried in a hot air oven (Memmert ULE500-Gemini-BV, Memmert GmbH + Co. KG, Schwabach, Germany) at 105 ± 1 °C, for 24 h. The moisture content was calculated using Equation (2):

(2) $$\%\mathrm{Moisture}=\frac{\left(\mathrm{Initial}\;\mathrm{weight}\;\mathrm{of}\;\mathrm{sample}\;-\;\mathrm{Final}\;\mathrm{weight}\;\mathrm{of}\;\mathrm{sample}\right)}{\mathrm{Initial}\;\mathrm{weight}\;\mathrm{of}\;\mathrm{sample}}\times 100$$

The water activity (a_w_) of the sample was determined in triplicate using a water activity meter (AquaLab series 3, Decagon Devices Inc., Pullman, WA, USA) at 25 °C.

#### 2.6.2. Texture Profile Analysis

The candy samples were examined (*n* = 5) for hardness (N) and springiness (%) using a texture profile analyzer (Texture Analyzer TA.XTplus, Stable Micro Systems, Surrey, UK), equipped with a P1 cylindrical probe (1-inch diameter). The sample was compressed with a 5 kg load cell with a pre-test speed of 1.00 mm.sec^−1^, test speed of 5.00 mm.sec^−1^, and post-test speed of 5.00 mm.sec^−1^, a strain of 50%, time of 15 sec, and trigger force of 5.0 g.

#### 2.6.3. Thiobarbituric Acid Reactive Substances (TBARS)

The lipid oxidation of the candy samples was examined in triplicate through the thiobarbituric acid reactive substances (TBARS) following the method of Pattarasiriroj et al. with slight modification [53]. Briefly, a 1 g sample was homogenized with 5 mL of 15% *w*/*v* Trichloroacetic acid (TCA), 0.375% Thiobarbituric acid (TBA), and 0.25 N Hydrochloric acid (HCL) for 30 s. The mixture was then heated in boiling water for 10 min and cooled in running tap water. After centrifuging at 3600× *g* at 25 °C for 20 min, the supernatant was collected and the absorbance at 532 nm was measured using a UV-Vis spectrophotometer (Genesys 10S UV-Vis, Madison, WI, USA). The standard curve of malonaldehyde in the concentration range of 0–2 ppm was constructed. The TBARS were expressed in mg malonaldehyde (MDA) per kg candy.

### 2.7. Statistical Analysis

The data were presented as the mean ± standard deviation. Using the SPSS software, a one-way analysis of variance (ANOVA) was performed (SPSS 23.0, SPSS Inc, Chicago, IL, USA). The significance of the difference was determined using Duncan’s Multiple Range Test at a 95% confidence level (*p* < 0.05).

## 3. Results

### 3.1. Film Characterization

#### 3.1.1. Film Thickness, and Mechanical Properties

Table 1 shows the thickness and mechanical properties in terms of the tensile strength (TS) and elongation at break (EAB) of the polypropylene (PP) and the rice starch-pectin biocomposite film with dragon fruit peel extract at 0 and 2% *w*/*v*, respectively. All three films had significant (*p* < 0.05) differences in thickness. The PP film was the thinnest. Concerning the biocomposite films, the 2% DPE-enriched film was thicker than the control (0% DPE) film. The polyphenols in DPE possibly contributed to the thickness of the film. The interactions through the non-covalent hydrogen bonding of the polysaccharide chain of rice starch-pectin biocomposite matrix with the polyphenols in the dragon fruit peel extract possibly led to the forming of the complex inclusion (V-type amylose) [54,55,56]. This helix structure of the polyphenols and polysaccharide chains of the starch-pectin biopolymer matrix limited the movement of the polysaccharide chain [57]. Thus, a more porous, coarser, and thicker structure of the 2% DPE-incorporated film was observed compared to the control (0% DPE) biopolymer film. Similar findings were reported in studies on the contribution of fiber addition to an increase in the thickness of starch-based films [57,58,59].

The tensile strength (TS) and the elongation at break (EAB) of all film samples were also examined, as shown in Table 1. The PP films exhibited drastically higher TS and EAB than the control film (0% DPE) and 2% DPE-enriched biocomposite films. The TS and EAB observed were inconsistent with a previous report [60]. As for the DPE-fortified biocomposite films, the TS and EAB of the 2% DPE-incorporated film sample were lower than those of the control (without DPE) film sample. The non-covalent bonds formed between polyphenols in DPE and the polysaccharide chain of the biopolymer matrix leading to the large inclusion possibly interfered with the inter hydrogen bonding between the plasticizer and amylose and amylopectin in the polysaccharide chains [61]. These weak intermolecular bonding led to a weaker film structure, thus reducing the tensile strength and flexibility of the DPE-incorporated film. Decreases in TS and EAB were noticed in cassava starch (CS) and sodium carboxymethyl cellulose (CMC) edible films with apple polyphenols [62] and in a rice starch-pectin composite film incorporating green tea extract [63]. A decreasing EAB was also observed in gelatin-based films with mango peel extract [27].

#### 3.1.2. Film Solubility, and Barrier Properties

Film solubility (FS) and water vapor permeability (WVP) values for all three film samples are exhibited in Table 1. While the least FS value was spotted in the PP film, all biocomposite (0%, 2% DPE) film samples exhibited significantly (*p* < 0.05) greater FS values than the PP film. The FS of the 2% DPE-enriched film was not significantly different from the control (without DPE) film, indicating that the FS of the 2% DPE-fortified biocomposite film samples was not altered by the addition of DPE. It is possible that at 2% *w*/*v* DPE, the biopolymer matrix could form non-covalent bonds creating an inclusion complex that could hold all added DPE molecules. Thus, there were no excess DPE molecules that could cause a fragile structure. So, the FS values were not significantly affected. However, when compared to the FS of a green tea extract-infused biocomposite film [63], the FS of all the DPE-enriched biocomposite films was significantly higher, which suggested that DPE-incorporated films might not be suitable for application in packaging high-moisture foods.

The WVP of the PP film was the smallest compared to the DPE-added biocomposite film. The 2% DPE-enriched film sample exhibited a significantly (*p* < 0.05) lower WVP than that of the 0% DPE (without DPE) film. The incorporation of the DPE altered the affinity to water of the resulting film. The forming of the V-amylose inclusion complex of polyphenol and polysaccharide chains might lead to the reduction of the availability of the hydrophilic exterior of the helix structure V-amylose [54,64]. Moreover, the hydrophobic interior of the helix inclusion also contributed to the reduction in water passage, resulting in the tortuous path of water permeation [65] in the DPE-enriched film. The formation of hydrogen bonds also limited molecular movement in the resulting film. Consequently, a reduction in WVP was observed. A similar decreasing trend in WVP was also reported in a potato starch film with tea polyphenols and MgO nanoparticles [66], gelatin-sodium alginate edible films with tea polyphenols [67], and a wheat starch-chitosan film with antioxidants [68].

#### 3.1.3. Film Appearance and Color

The appearance and color values of the PP, control (0% DPE), and 2% DPE biocomposite films are illustrated in Table 2. The PP film was clear, while the control (without DPE) biocomposite film was opaque white. The 2% DPE-incorporated film appeared in deep purplish red, which was attributed to the betacyanin present in the DPE. The 2% DPE-enriched film sample was the darkest (*p* < 0.05) compared to the PP and control (0% DPE) film samples, as the L* value was at the lowest value. The 2% DPE-enriched biocomposite film had the highest a* value (*p* < 0.05), which corresponds to the betacyanin compound in DPE. The b* and DE* of the 2% DPE-fortified film sample were also the highest (*p* < 0.05) among all samples. The addition of DPE considerably enhanced the color changes toward red. A similar red color trend was reported in the incorporation of dragon fruit peel extract into Cassava Starch–Chitosan [28] and κ-carrageenan-based pH-sensing films [69].

#### 3.1.4. Determination of Total Phenolic Content and Total Betacyanins Content

Table 3 shows the total phenolic content (TPC) and total betacyanins content (TBC) in the film samples. Phenols and betacyanins were not detected in the PP and 0% DPE (without DPE) biocomposite film samples. While the 2% DPE-enriched film sample exhibited phenol and betacyanin content. This incidence was attributed to the DPE [34,70] incorporation into the biocomposite film [28,69].

#### 3.1.5. Antioxidant Activities

The DPPH radical scavenging activity and ferric-reducing antioxidant power (FRAP) of all film samples are illustrated in Table 3. The PP and 0% DPE (without DPE) film samples exhibited no antioxidant activities. While the 2% DPE-fortified biocomposite film showed FRAP and DPPH antioxidant activities, corresponding to the TPC and TBC. The DPE contributed to the antioxidant capacity of the 2% DPE-incorporated biocomposite film [70]. Food packaging films with antioxidant ability may minimize lipid oxidation in fat-containing food products, thus preserving the quality of food products [71,72].

#### 3.1.6. Film Morphology

**Figure 1:**
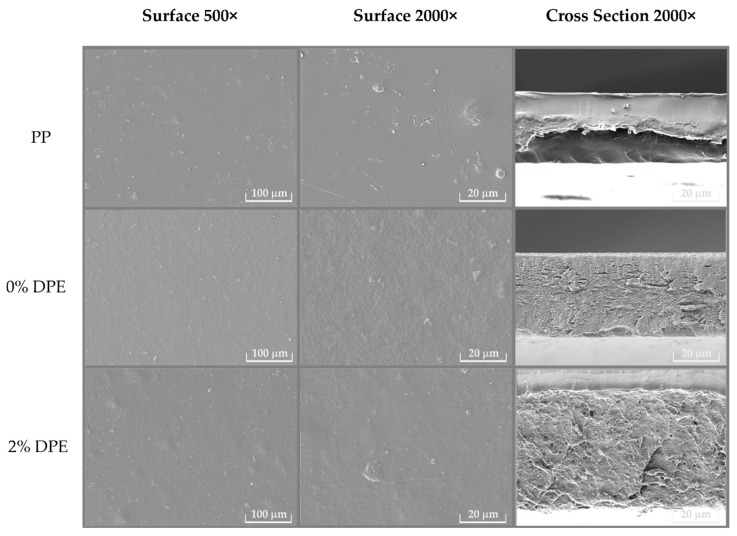
FESEM micrographs of surface (magnification: 500× and 2000×) and cross-section (magnification: 2000×) of polypropylene (PP), 0% DPE: 0% dragon fruit peel extract-enriched biocomposite films (% *w*/*v*), 2% DPE: 2% dragon fruit peel extract-enriched biocomposite films (% *w*/*v*).

Figure 1 shows FESEM micrographs of the surface at 500× and 2000× magnification and the cross-section at 2000× magnification of all film samples. The PP film sample exhibited a smooth and homogeneous surface. The cross-sectional micrograph of the PP film was compact and flat. Conversely, a heterogeneous and rough surface and cross-section were observed with the 0% DPE (without DPE) and 2% DPE-enriched biocomposite films. Though the DPE was evenly dispersed in the biocomposite matrix, the resulting 2% DPE-fortified film revealed coarser and more tortuous channels on the cross-sectional micrograph. The protrusion and roughness spotted on the surface, and cross-sectional areas of the DPE-enriched film are possibly caused by the DPE incorporation, associated with the increase in thickness and decrease in WVP of the resulting 2% DPE-enriched biocomposite film compared to the 0% DPE (without DPE) film sample. An effect due to DPE incorporation on film morphology has also been exhibited in a pH-sensitive intelligent gelatin-based film [73] cassava starch–chitosan film [28]. In addition, the effect of substrate surface modification was observed in other natural active ingredients, such as cinnamaldehyde [74], and sugarcane purple rind extract [75], on the surface of carbon steel and steel, respectively.

### 3.2. Quality Attributes of Coconut Milk Candy

#### 3.2.1. Moisture Content and Water Activity

**Figure 2:**
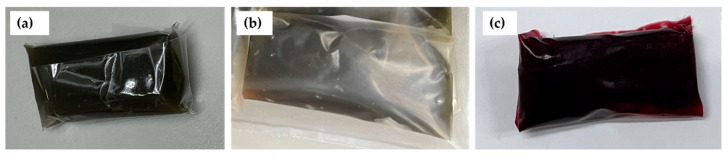
Coconut milk candy wrapped in PP film (**a**), Control (0% DPE) biocomposite film (**b**), and 2% DPE-enriched biocomposite film (**c**).

**Figure 3:**
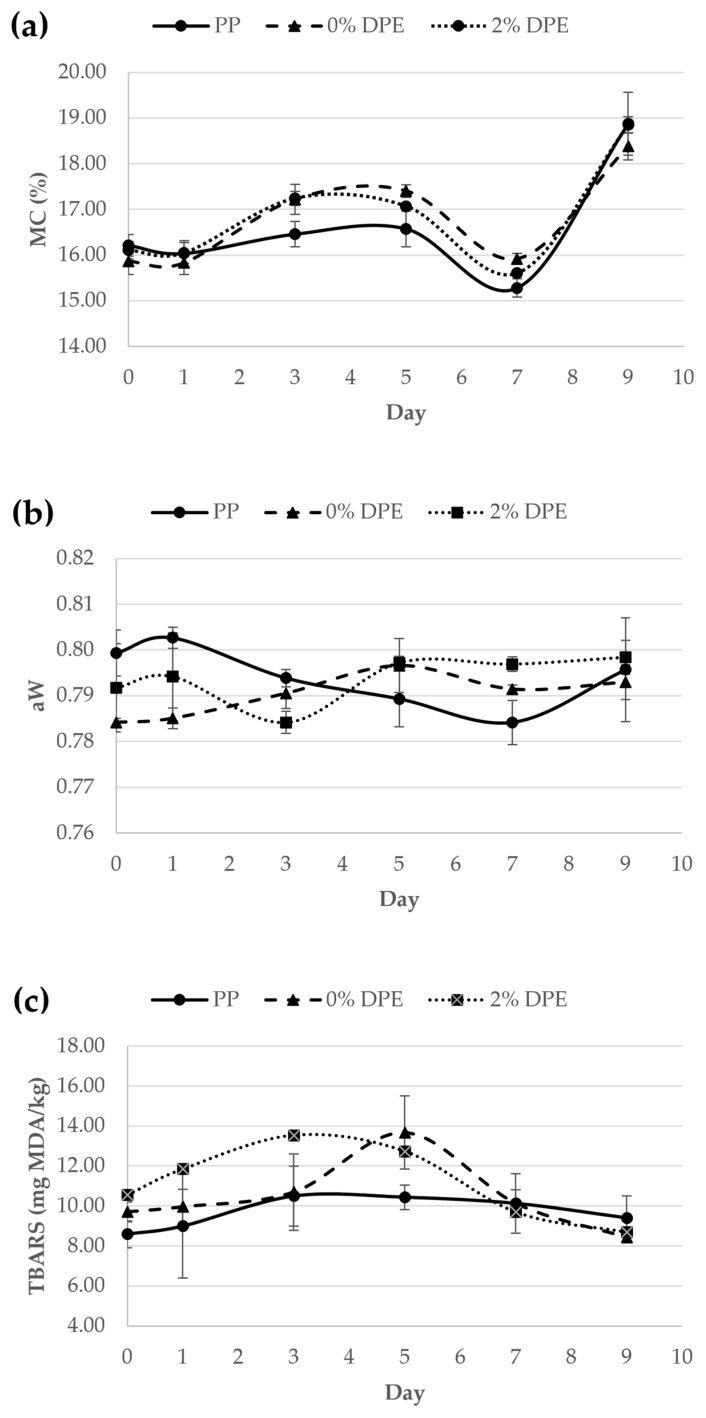
Moisture content (**a**), water activity (**b**), and Thiobarbituric acid reactive substances (TBARS) (**c**) of coconut milk candy wrapped in polypropylene (PP), 0% DPE biocomposite film, and 2% DPE-enriched biocomposite films (% *w*/*v*).

Figure 2a–c show the coconut milk candies wrapped in the PP, 0% DPE, and 2% DPE-enriched films, respectively. The typical coconut milk candy had a dark brown color due to the caramelization of sugar during the candy-making process [40,76]. Figure 3a–c. illustrate the moisture content (MC), the water activity(a_w_), and the thiobarbituric acid reactive substances (TBARS) of the coconut milk candy wrapped in the PP, 0% DPE, and 2% DPE-incorporated films and kept at 30 ± 1 °C, 75% RH. The quality attributes of the coconut milk candies wrapped in different film samples were monitored at day 0, 1, 3, 5, 7, and 9, respectively.

The moisture content (MC) values indicated the total amount of water in the food product. While the water activity (a_w_), indicated the free water in the food product that the microorganism and biochemical/chemical reactions can utilize. Higher a_w_ implied a higher rate of quality changes. The a_w_ is typically expressed as the ratio of a partial water vapor pressure of the food product to that of pure water [77]. MC and a_w_ are related to the quality changes in the food product. Coconut milk candy is an intermediate moisture food (MC 15–40%, a_w_ 0.70–0.85), which makes it vulnerable to food deterioration, such as chemical reactions, and microorganism growth, resulting in a short shelf-life [78]. Though coconut milk candy is generally considered microbiologically stable at room temperature, mold and yeast may still grow [79]. Covering the coconut candy in different types of wrapping films (i.e., PP, 0% DPE, 2% PDE) did not significantly (*p* < 0.05) alter the moisture content (15.83–18.88%), and the free water activity (0.784–0.803) of the coconut milk candy over the storage period of 9 days. This could be attributed to the limited number of available -OH groups in the phenolic compound in DPE to bind free water in the coconut candy as they possibly formed hydrogen bonds with the biocomposite matrix in the film [79]. However, on day 9, the moisture content of all candy samples wrapped in all film samples increased significantly compared to day 0. The rise in MC over a long period of storage at high relative humidity might be attributed to starch retrogradation [40]. The reassociation of amylopectin in the candy contributed to the squeezing out of water molecules during storage. The moisture content is also related to the change in textural properties during the storage of the candy, as the small molecules of water can migrate during the early stage of storage [80].

#### 3.2.2. Thiobarbituric Acid Reactive Substances

The lipid oxidation revealed by the thiobarbituric acid reactive substances (TBARS) assay of all wrapped coconut milk candies was monitored over 9 days. TBARS reacts with the secondary oxidative products, mainly malonaldehydes (MDA), and generates a pink chromogen that can be measured at 532–535 nm [77]. As shown in Figure 3c, the TBARS values (mg MDA/kg) of all candy samples increased from day 0 to day 5, suggesting that the candy had undergone lipid oxidation. However, after day 5, all candy samples showed a decreasing trend in TBARS values. This phenomenon possibly indicated that all lipid substrates in the coconut milk candy were completely oxidized. No delay in the lipid peroxidation in candy wrapped in the 2% DPE-enriched film was observed. The data obtained did not show different trends for different film types, which could be attributed to the complex nature of lipid-starch inclusion [81]. Since all candy samples wrapped with various film types were not tightly sealed, the oxygen and moisture might percolate through the edge of the wrapping film [82]. In addition, the phenolic compounds in the DPE bound to the film matrix might not be able to interfere with the propagation of lipid oxidation. Thus, further investigation on the major candy quality deterioration mechanism should be performed.

#### 3.2.3. Texture Profile Analysis

Texture plays an important role in coconut milk candy [37]. Table 4 shows the texture profile analysis regarding hardness (N) and springiness (%). Hardness reflected the force required to bite the candy sample. The hardness of all candies wrapped with different film samples increased over the nine-day aging, possibly owing to starch retrogradation and/or the formation of amylose-lipid complex [40,81]. The coconut milk candy contained myristic fatty acid present in coconut milk and possessed a high ability to form amylose-lipid complex [81]. The reassociation of amylopectin, the main composition in the glutinous rice flour used in producing the coconut milk candy, also resulted in texture hardening over storage [39]. The coconut milk candy enveloped in the 0% DPE (without DPE) biocomposite film exhibited significantly (*p* < 0.05) higher texture hardening than those covered in the PP and 2% DPE-enriched films, suggesting that more starch retrogradation had occurred [83]. The hardness of candy wrapped in PP film was not significantly altered. According to the FESEM micrographs (Figure 1), the PP film showed a smooth and compact structure, while the 2% DPE-fortified film exhibited a thick and tortuous path, leading to a lower rate of starch retrogradation due to the limiting of water passage through the film. This occurrence corresponded to the lower WVP values of PP and 2% DPE-incorporated film than that of the control (0% DPE) biocomposite film (Table 1).

Springiness (%) relates to the rebounding of the sample after being bitten (deformed). The springiness values of the coconut candies wrapped in the PP, 0% DPE, and 2% DPE-enriched biocomposite films were not affected over the nine-day storage. Short-chain amylopectin and amylose may leach out from the starch granule during the heating process during candy production [84], forming a starch-lipid complex through the hydrophobic end of the amylose single helix. This phenomenon contributed to a stronger and more stable starch V-type crystalline network [81], resulting in stable springiness of the coconut milk candy over storage.

## 4. Conclusions

In this study, rice starch-pectin biocomposite films enriched with dragon fruit peel extract (0% DPE (control) and 2% DPE (% *w*/*v*)) were applied as coconut milk candy wrapping films and compared to candy wrapped in polypropylene (PP) film (commercial coconut milk candy wrapping film). The PP film exhibited a thin, clear, compact, and dense microstructure, the lowest film solubility, lowest WVP, highest tensile strength, and elongation at break compared to the 0% DPE and 2% DPE-fortified biocomposite films. However, the antioxidant and antioxidant scavenging activity were not observed in the PP film or the control (0% DPE) biocomposite film. The purplish-red 2% DPE-enriched biocomposite film was the only film containing the phenolic compounds and showing antioxidant ability. FESEM micrographs showed the rough surface and porous path of all biocomposite films. Regarding the application of the films as candy wrappers, the quality attributes of the coconut milk candy were inspected over nine-day storage. The obtained results revealed that the moisture content and the water activities of all candies wrapped in different film samples were not significantly (*p* < 0.05) altered over the studied period. The TBARS of all candy samples was increased over the first five days of storage. The hardness of all candy samples increased over the studied period. The coconut candy in the 0% DPE-enriched biocomposite film exhibited significantly higher hardness after day 5. However, no significant changes in springiness were observed in any of the coconut candy samples. The mechanisms of quality deterioration of the coconut milk candy were intricate; therefore, further investigation on the potential methods to delay deterioration factors and to extend the shelf-life of coconut milk candy should be carried out.

## Figures and Tables

**Table 1:** Thickness, film solubility, mechanical, and barrier properties of polypropylene and DPE-enriched biocomposite films.

Film Sample (%, *w*/*v*)	Thickness (mm)	TS (MPa)	EAB (%)	FS (%)	WVP $\left(\times 10^{-11}\;g\;m/m^{2}\;s\;\mathrm{Pa}\right)$
PP	0.036 ± 0.003 ^c^	42.91 ± 4.35 ^a^	606.08 ± 88.77 ^a^	39.50 ± 7.48 ^b^	0.08 ± 0.01 ^c^
0% DPE	0.049 ± 0.002 ^b^	5.72 ± 1.42 ^b^	28.06 ± 1.41 ^b^	74.31 ± 2.94 ^a^	9.57 ± 1.13 ^a^
2% DPE	0.075 ± 0.001 ^a^	1.23 ± 0.38 ^c^	25.28 ± 1.19 ^c^	76.00 ± 3.22 ^a^	5.22 ± 0.22 ^b^

Values are given as mean ± SD from *n* = 5 determination for thickness; *n* = 7 for determinations of TS and EAB; *n* = 3 for determinations of FS, and WVP. Different superscripts in each column are significantly different (*p* < 0.05). PP: polypropylene, DPE: dragon fruit peel extract (% *w*/*v*), FS: film solubility, TS: tensile strength, EAB: elongation at break, and WVP: water vapor permeability.

**Table 2:** Appearance and color values of polypropylene and DPE-enriched biocomposite films.

Film Sample (%, *w*/*v*)	Appearance	L*	a*	b*	ΔE*
PP	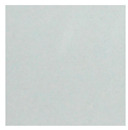	82.60 ± 0.34 ^b^	0.62 ± 0.01 ^b^	−6.74 ± 0.06 ^c^	34.94 ± 0.15 ^b^
0% DPE (Control)	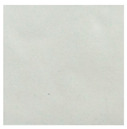	83.09 ± 0.13 ^a^	0.37 ± 0.01 ^c^	−3.04 ± 0.13 ^b^	32.53 ± 0.16 ^c^
2% DPE	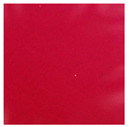	34.48 ± 0.21 ^c^	49.87 ± 0.25 ^a^	8.54 ± 0.22 ^a^	50.37 ± 0.21 ^a^

Values are given as mean ± SD from *n* = 3. Different superscripts in each column are significantly different (*p* < 0.05). PP: polypropylene, DPE: dragon fruit peel extract (% *w*/*v*).

**Table 3:** Bioactive compound and antioxidant properties of the films.

Film Sample (%, *w*/*v*)	TPC (mg GAE/g)	TBC (mg/g)	FRAP (mM Fe (II)/g)	DPPH (mM Trolox/g)
PP	ND	ND	ND	ND
0% DPE	ND	ND	ND	ND
2% DPE	18.18 ± 0.78 ^a^	0.91 ± 0.01 ^a^	30.08 ± 2.68 ^a^	194.50 ± 7.43 ^a^

Values are given as mean ± SD from *n* = 3, Different superscripts in each column are significantly different (*p* < 0.05). PP: polypropylene, DPE: dragon fruit peel extract (% *w*/*v*), TPC: total phenolic content, TBC: total betacyanins content, FRAP: ferric reducing antioxidant power, and DPPH: (2,2-diphenyl-1-picryhydrazyl) radical scavenging activity.

**Table 4:** Texture profile analysis of coconut milk candy wrapped in polypropylene and DPE-enriched biocomposite films.

Day	Hardness (N)			Springiness (%)		
	PP	0% DPE	2% DPE	PP	0% DPE	2% DPE
0	5.57 ± 0.86 ^eA^	6.36 ± 1.33 ^cA^	6.54 ± 1.71 ^cA^	92.75 ± 4.19 ^aA^	93.65 ± 4.69 ^aA^	90.06 ± 3.26 ^bA^
1	7.30 ± 0.75 ^dA^	6.67 ± 1.06 ^cA^	5.91 ± 1.18 ^cA^	95.91 ± 3.22 ^aA^	94.53 ± 2.81 ^aA^	96.97 ± 4.77 ^abA^
3	9.34 ± 0.89 ^cdA^	6.82 ± 1.32 ^cB^	6.80 ± 2.22 ^bcAB^	95.22 ± 4.44 ^aA^	97.88 ± 2.72 ^aA^	97.91 ± 3.63 ^aA^
5	8.28 ± 1.54 ^cA^	10.21 ± 1.71 ^bA^	10.24 ± 1.58 ^bA^	94.11 ± 3.54 ^aA^	94.31 ± 0.97 ^aA^	99.42 ± 5.10 ^aA^
7	13.78 ± 2.69 ^bB^	23.65 ± 2.88 ^aA^	17.52 ± 2.63 ^aAB^	96.90 ± 3.86 ^aA^	97.28 ± 5.24 ^aA^	98.96 ± 3.53 ^aA^
9	19.71 ± 2.53 ^aB^	27.15 ± 2.98 ^aA^	16.62 ± 1.14 ^aB^	95.53 ± 4.16 ^aA^	95.94 ± 3.53 ^aA^	99.9 ± 1.34 ^aA^

Values (*n* = 5) are given as mean ± SD. Different letters indicate significantly different (*p* < 0.05). Different small letter superscripts in each column are significantly different (*p* < 0.05). Different capital letter superscripts in each row indicate significant differences (*p* < 0.05). Texture profile analysis of coconut milk candy wrapped in the polypropylene (PP) film, 0% DPE: 0% dragon fruit peel extract-enriched biocomposite films (% *w*/*v*), 2% DPE: 2% dragon fruit peel extract-enriched biocomposite films (% *w*/*v*).

## Data Availability

The data presented in this study are available on request from the corresponding author.
